# Systematic Review of the Efficacy of Cognitive-Behavior Therapy Related Treatments for Victims of Natural Disasters: A Worldwide Problem

**DOI:** 10.1371/journal.pone.0109013

**Published:** 2014-10-08

**Authors:** Alessandra Pereira Lopes, Tânia Fagundes Macedo, Evandro Silva Freire Coutinho, Ivan Figueira, Paula Rui Ventura

**Affiliations:** 1 Institute of Psychiatry, Universidade Federal do Rio de Janeiro, Rio de Janeiro, Brazil; 2 D'Or Institute for Research and Education, Rio de Janeiro, Brazil; 3 Department of Epidemiology, Escola Nacional de Saúde Pública, Rio de Janeiro, Brazil; 4 Psychological Institute, Universidade Federal do Rio de Janeiro, Rio de Janeiro, Brazil; University of California, San Francisco, United States of America

## Abstract

Natural disasters can have devastating consequences. Each year, about 225 million people are victims of natural disasters worldwide, and up to 13,5 million of these people can develop post-traumatic stress disorder (PTSD) in the first or second year following the disaster. Cognitive-behavior therapy (CBT) is the first-choice treatment for this disorder. In order to evaluate the efficacy of psychotherapeutic treatment based on cognitive-behavior therapy for people who developed post traumatic stress disorder after natural disasters we conducted a systematic search of published studies. We used the terms reported below in the electronic databases ISI Web of Science, PsycINFO, PubMed, PILOTS and Scopus with no restrictions of language or publication date. Articles that described randomized controlled, non-randomized controlled and non controlled studies on the efficacy of cognitive-behavior therapy for individuals diagnosed with post-traumatic stress disorder after exposure to a natural disaster were eligible for inclusion. The studies were required to use a standardized measure of effectiveness before and after the intervention and have a group of patients who had used cognitive-behavior therapy as the only intervention. Our search identified 820 studies, and 11 were selected for this review. These 11 studies involved 742 subjects, 10 related to earthquakes and 1 to a hurricane. The cognitive-behavior therapy techniques used were various: 7 studies used exposure therapy, 2 studies used problem solving, and the only 2 studies with adolescents used techniques including reconstructions and reprocessing of the traumatic experience. As limitations, the search involved only five electronic databases, no experts in the field were consulted, and the heterogeneity of the findings made it impossible to perform a meta-analysis. The results suggest the efficacy of cognitive-behavior therapy, particularly exposure techniques, for the treatment of post-traumatic stress disorder after earthquakes. However, further studies with stronger methodologies, i.e. randomized-control trials and non-randomized controlled trials, are needed.

## Introduction

The consequences of natural disasters can be devastating, as they are large-scale, potentially traumatic events which affect a significant number of people [1]. Along with the increase of the world population between 1960 and 2005, an annual increase of 5% in the number of natural disasters and of 4% in the number of people affected by these disasters was observed [2].

About 225 million people worldwide are affected by natural disasters annually, i.e. 1/25 people in the world [3]. Of these, up to 13,5 million (3–6% of 225 million exposed to this kind of disaster) can develop post-traumatic stress disorder (PTSD) in the first or second year following the disaster [4].

Post-traumatic stress disorder (PTSD) can occur in people who have experienced, witnessed or known about a traumatic event that posed risk of death, serious harm or threat to physical integrity, and as a response to it have felt intense fear, helplessness or horror. The symptoms usually appear one month after the trauma and involve three clusters: re-experiencing, avoidance and hyperarousal [5].

For treatment of PTSD, first-choice interventions are cognitive-behavioral therapy (CBT) and drug therapy [6]. CBT includes exposure techniques, cognitive re-structuring and anxiety management [7]–[8]. The exposure technique is the most used and most successful technique for treating this disorder. It involves patients confronting their fears, either through imagination or in vivo. Anxiety management includes relaxation training, diaphragm breathing, social skills training, and distraction techniques. In a literature review, Foa and Meadows [7] showed that prolonged exposure and stress inoculation training are effective in reducing PTSD symptoms.

International guidelines advise using CBT a few weeks after a disaster or other shocking event to reduce PTSD symptoms [9]. However, despite the increase in both the number and severity of natural disasters [10] and the significant impact on health, the disaster-related literature on PTSD is surprisingly scanty as regards the assessment of efficacy of interventions for this type of disorder [11]. The aim of this study is to evaluate the efficacy of psychotherapeutic treatment based on CBT for individuals who developed PTSD after natural disasters, through a systematic review. To the best of our knowledge there is no systematic review or meta-analyses about CBT efficacy for PTSD after natural disasters.

## Methods

### Search Strategy

We conducted systematic searches in the electronic databases ISI Web of Science, PsycINFO, PubMed, PILOTS and Scopus with no restrictions of language or publication date. The search occurred until April 03, 2013. We performed advance searches in all 5 databases using the following search terms:

- PTSD OR “stress disorder” OR “Post-traumatic Stress Disorder”
- earthquake OR “mass trauma” OR disaster OR flood OR tsunami OR cyclone OR hurricane OR whirlwind OR flaw OR “vortex typhoon” OR tornado OR “natural disaster*”
- “behavi* therapy” OR “cognitive therapy” OR “cognitive behavio* therapy” OR “exposure therapy” OR “exposure treatment” OR “exposure session*” OR CBT OR “cognitive reest*” OR “anxiety manage*” OR flooding OR “systematic desens*”

The terms indicated in each item were combined using “AND”, a function which is available in all databases. In the ISI Web of Science the search was restricted to articles and notes, but in PsycINFO, PubMed, PILOTS and Scopus it was conducted in all fields.

Besides these searches in electronic databases, we also performed manual searches in the reference sections of published texts in the field as an additional guarantee that every eligible article for inclusion was indeed covered by the survey.

**Figure 1:**
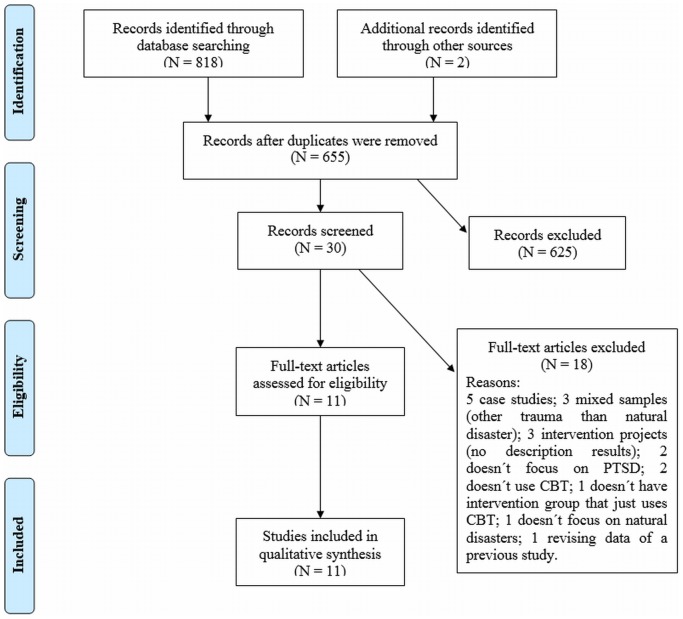
Flowchart of the process of identification and selection of studies.

We then analyzed the abstracts of all studies identified and excluded papers that did not meet the selection criteria. If a given abstract met inclusion criteria for the study, the full text was analyzed [12]. The selection process is described in Figure 1.

For this research, a wide definition of CBT was considered, encompassing behavioral and/or cognitive strategies.

### Criteria for including studies in this review

We selected articles that reported randomized controlled, non-randomized controlled or non controlled studies using CBT for individuals diagnosed with PTSD after exposure to a natural disaster, and which provided a standardized measure of effectiveness before and after the intervention. To distinguish non controlled studies from case studies, we followed the criterion that open trials should comprise 10 cases or more [13].

We excluded review articles, book chapters, dissertations and theses, as well as studies without a standardized measure of effectiveness before and after the intervention, case studies, studies without patients using CBT as the only intervention; studies of samples with diagnosis of PTSD resulting from trauma other than a natural disaster; studies with animal models; and studies without a formal diagnosis of PTSD.

### Quality evaluation of the studies

After the search phase, we did an analysis of the methodological quality of the randomized controlled trials and the non-randomized studies selected based on an adaptation of the Cochrane Collaboration Tool for Assessing the Risk of Bias [14]. One author (A.L.) extracted study characteristics and another author (E.C.) assessed risk of bias (Table 1).

**Table 1:** Description of variables in evaluation of methodological quality.

Criterion	Description	Evaluation
Random Sequence Generation	Evaluate the way the randomization sequence was generated	Low Risk of Bias: proper randomized way of generating randomization sequence; examples are sequences generated by computer programs
		Unclear Risk of Bias: Does not inform how the randomization sequence was generated
		High Risk of Bias: Non-randomized or inadequate way of generating randomization sequence
Allocation Concealment	Evaluate concealment of sequence, i.e. how difficult it was for participants or evaluators to predict to which group the next subjects would be assigned	Low Risk of Bias: The randomization sequence was generated so as to promote concealment of it
		Unclear Risk of Bias: Does not inform how randomization sequence and attempt of concealment was generated.
		High Risk of Bias: No attempt of concealment of randomization sequence
Single Blinding (rater)	Evaluate if the evaluator was “blind”, i.e. when evaluating he/she was unaware of which group the subject belonged to (intervention or waiting list)	Low Risk of Bias: Evaluator did not know which group the subject belonged to while evaluating
		Unclear Risk of Bias: Does not inform if evaluator was aware of the group the subject belong to
		High Risk of Bias: Evaluator knew which group the subject was assigned to.
Incomplete Outcome Data	Evaluate information about loss of data at follow-up	Low Risk of Bias: There was little or no loss along the study, and this is mentioned by the author
		Unclear Risk of Bias: No losses were mentioned, when the numbers clearly pointed so
		High Risk of Bias: Great loss along the study
Selective Reporting (Reporting Bias)	Mention along the results all of the criteria, variables and measures described in the aim and methods of the study	Low Risk of Bias: Results of all the criteria, variables and measures mentioned
		Unclear Risk of Bias: No information about the criteria, variables and measures used, as well as their results.
		High Risk of Bias: Criteria, variables and measures were used that were not reported in the results section
Concomitant Treatment	Inform if there was treatment concomitant to the provided one	Low Risk of Bias: Absence of concomitant treatment
		Unclear Risk of Bias: No information about concomitant treatment
		High Risk of Bias: Presence of concomitant treatment to the one offered in the study
Description of Treatment	Describe activities performed during the treatment	Low Risk of Bias: Mentions how many sessions were held and activities were described by session
		Unclear Risk of Bias: Incomplete description about the treatment
		High Risk of Bias: Did not provide description of the number and content of sessions.
Description of Control Activity	Describe activities performed by the control group or waiting list	Low Risk of Bias: Mentions if control group performed any activities, and if so, which ones
		Unclear Risk of Bias: Incomplete description of activities performed by control group
		High Risk of Bias: No information on activities performed by control or waiting list group. Or performed some similar activity to the intervention group
Identification of Internal Comparability	Identify numerical or social demographic differences between intervention and control groups	Low Risk of Bias: No differences found between the groups and authors identified and highlighted such differences
		Unclear Risk of Bias: It was not possible to clearly evaluate the differences between groups
		High Risk of Bias: There were differences but authors do not mention it
Statistical Treatment for Internal Comparability	Use statistical treatment to control for differences observed between the groups (intervention X control)	Low Risk of Bias: There was difference between the groups and a statistical method was used to control for it
		Unclear Risk of Bias: Authors do not provide data for this evaluation
		High Risk of Bias: A difference was found, but no statistical treatment was used to control for it.

## Results

Our survey identified 820 studies, from which 11 were selected for this review (Figure 1) according to the above-mentioned inclusion criteria: 3 randomized controlled, 3 non-randomized controlled and 5 non controlled trials. Two of these studies used the DSM-III-R (Diagnostic and Statistical Manual of Mental Disorders – Third Edition Revised) as a basis for diagnosing PTSD [15], [16], while the other ones used the fourth edition. Table 2 shows the descriptions of the studies.

**Table 2:** Description of studies.

Study	Sample	Trauma	Blinding	Type of Intervention	Number of Sessions	Assessment of PTSD Symptoms	Moments of evaluation	Results in PTSD symptoms
**Randomized Controlled Studies**								
Basoglu *et. al.*, 2005	59 adults (31 to SSBT and 28 to WL)	Earthquake	Double Blinding (Assessments + Participants)	Behavioral Treatment/	1 session of 60 minutes	CAPS	Before and week 6, 12, 24 and 1, 2 year after treatment	Treatment was effective in reducing PTSD symptoms (P<.001). 71% of the sample had a reduction in PTSD symptoms 24 weeks after the intervention.
				Self-exposure				
Basoglu *et. al.*, 2007	31 adults (16 to SSBT and 15 to RA)	Earthquake	Double Blinding (Assessments + Participants)	Behavioral Treatment/	1 session of 60 minutes	CAPS	Before and week 4, 8, 12, 24 and 1, 2 year after treatment	Treatment effects were significant (P<0.01), 80% of the sample had significant reduction in PTSD symptoms and fear 24 weeks after the intervention. Between-group comparisons were significant on all measures.
				Exposure				
Zang *et. al.*, 2013	22 adults (11 to NET and 11 to WL)	Earthquake	Single Blinding (Assessment)	NET	4 sessions of 90 minutes	IES-R	Before and immediately after treatment and 2 weeks and 2 months after treatment	The participants that received NET showed significant (P<0.01) reductions in PTSD symptoms immediately after the treatment.
**Non-randomized Controlled Studies**								
Goenjian *et. al.*, 1997	64 adolescents (35 to group treatment and 29 to control group)	Earthquake	Not Available	Brief trauma/grief focused psychotherapy	4 group sessions of 30 minutes and two individual sessions of 60 minutes	CPTSD-RI	Before and after treatment	After intervention the adolescents that received treatment had a significant (P<0.05) decrease in all three categories of PTSD (re-experiencing, avoidance and hyper arousal autonomic)
Goenjian *et. al.*, 2005	63 adolescents (36 to group treatment and 27 to control group)	Earthquake	Not Available	Brief trauma/grief focused treatment	4 group sessions of 30 minutes and two individual sessions of 60 minutes	CPTSD-RI	Before and after treatment	Both groups had significantly decreased scores (P<0.001), but in the treated group all three categories (re-experiencing, avoidance and hyper arousal autonomic) decreased, whereas in the untreated group only re-experiencing symptom was reduced.
Ferdos&Seyed-Hossein, 2007	160 adults (80 to experimental group and 80 to control group)	Earthquake	Not Available	Problem Solving	12 sessions of 2 hours	Mississippi PTSD Scale	Before and after treatment	There was significant (P = 0.001) reduction of PTSD symptoms compared to control groups. The experimental groups showed significantly (P<0.02) increased coping skills focused on problem solving.
**Not Controlled Studies**								
Basoglu *et. al.*, 2003	167 adults	Earthquake	Not Applied	Behavioral Treatment/Self-exposure	An average of 3 to 4 sessions	TSSC	Before and after treatment, and follow-up at 1 to 2, 2 to 3 and 3 to 9 months after the treatment	76% of participants had a reduction in PTSD symptoms with one session and 88% with two sessions, so the treatment had a significant (P<0.001) effect in reducing PTSD symptoms
Giannopoulou *et. al.*, 2006	20 children	Earthquake	Not Applied	CBT	6 sessions of 2 hours with children and 1 session with parents	CRIES	Before and immediately after the treatment and 18 months and 4 years after the intervention	Treatment caused significant (P = 0.001) reduction in PTSD symptoms. Of 17 children who completed treatment, only two continued to meet PTSD diagnosis, i.e. the others showed remission of the disorder.
Oflaz *et. al.*, 2008	14 adults	Earthquake	Not Applied	Psychoeducation and Problem Solving	6 sessions of 60 to 90 minutes	CAPS	Before and after the treatment	A significant (P = 0.001) reduction of PTSD symptoms was observed in psychoeducation and problem solving group.
Jaycox *et. al.*, 2010	118 children (58 to CBITS and 60 to TF-CBT)	Hurricane	Not Applied	CBT	CBITS = 10 group sessions and 1 to 3 individual sessions	CPTSD-SS	Before and 5, 10 months after the treatment	Both groups showed significant reduction in PSTD symptoms (CBITS = P<0.001 and TF-CBT = P<0.01).
					TF-CBT = 12 individual or conjoint sessions (children and parents)			
Zhang *et. al.*, 2011	24 persons (aged 6–80 years)	Earthquake	Not Applied	CBT	30 minutes of daily sessions for 1 week	Chinese version of IES-R	Before and after treatment	Participants that received CBT and CBT plus acupoint stimulation showed significant (P<0.01) reductions of PTSD symptoms after treatment.

The studies of Zhang et. al. (2011), Jaycox et. al. (2010) and Oflaz et. al. (2008) are originally randomized studies, but for the purposes of this review and analysis of their results they were used as open trials. Abbreviations: IES-R = Impact of Event Scale – Revised; CPTSD-RI = Child Posttraumatic Stress Disorder Reaction Index; CAPS = Clinician-Administered PTSD Scale; CPTSD-SS = Child PTSD Symptom Scale; CRIES = Children's Revised Impact of Event Scale; TSSC = Traumatic Stress Symptom Checklist; SSBT = Single Session Behavioral Treatment; WL = Waiting List; RA = Repeated Assessments; NET = Narrative Exposure Therapy; CBITS = Cognitive-Behavioral Intervention for Trauma in Schools; TF-CBT = Trauma-Focused Cognitive-Behavioral Therapy; CBT = Cognitive-Behavioral Therapy.

We excluded 165 duplicated studies that had been found in the electronic search in the five databases. After reading the abstract, 625 studies were excluded for the following reasons: 10 were case studies, 111 were reviews, 47 were book chapters, dissertations or theses, 2 did not have an intervention group that used only CBT, 146 did not focus on treatment response, 67 did not focus on PTSD, 237 did not focus on natural disasters, 3 did not use humans and 2 had the aim of validating instruments. From the remaining 30 studies, 18 were excluded after reading the full text for the following reasons: 5 were case studies, 3 used mixed samples, 3 were intervention projects (had no results), 2 did not focus on PTSD, 2 did not use CBT, 1 did not have an intervention group that used only CBT intervention, 1 did not focus on natural disasters, and 1 revised data from a previous study.

Of the 11 articles, 7 used exposure techniques (three randomized studies [17]–[19] and four non controlled studies [20]–[23]), and of these, 2 focused on exposure through narrative [19], [23] and 2 used exposure with other cognitive and behavioral techniques, which were also the two studies with children [21], [22]. Two other trials used problem solving techniques [24], [25]. The only two studies with adolescents [15], [16] used techniques encompassing reconstruction and reprocessing the traumatic experience, identification of traumatic memories and building up tolerance to related reactions, development of acceptance and adaptation to earthquake-related changes, mental reconstruction of deceased persons, and fostering of normal development.

**Figure 2:**
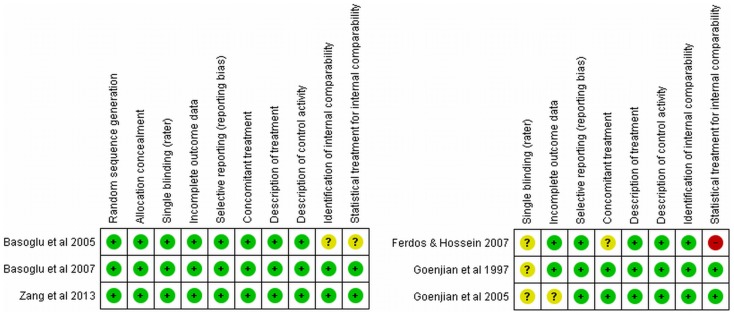
Methodological Analysis for Study.

**Figure 3:**
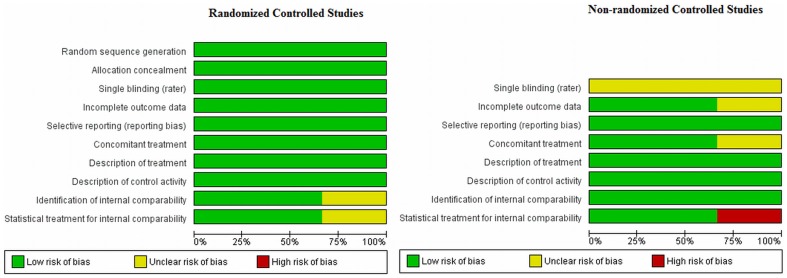
Methodological Analysis by Criteria.

Figures 2 and 3 summarize the different aspects concerning the methodological quality of the studies. Among the randomized clinical trials most of the methodological aspects were well covered. Only one study did not mention if the compared groups were balanced concerning age, gender and other potential confounders. One study did not mention if patients received other treatments besides the intervention and another one did not control for potential confounders. All non-randomized studies did not mention the use of blinding assessment.

### Randomized Controlled Studies

Basoglu, Salcioglu, Livanou, Kalender and Acar [17] tested the effectiveness of a behavioral treatment session as compared to waiting list three years after the earthquake that hit Turkey in 1999. The intervention focused on reduction of fear and avoidance, habituation to anxiogenic stimulus, and increasing in the sense of control over traumatic stressors. The first stage of treatment involved the identification of problems like fear and avoidance of earthquakes, re-experiencing and hyperarousal. The second stage consisted of explaining the mechanism and logic of exposures. The final step in the session set up goals (stimuli related to the trauma) and instructions for self-exposure. No cognitive re-structuring was conducted during the treatment. The study found that a single session of behavioral therapy was a cost-effective strategy for earthquake survivors with PTSD, particularly those of lower socioeconomic status (SES) and education.

Basoglu, Salcioglu and Livanou [18] examined the efficacy of a single exposure session in an earthquake simulator and self-exposure instructions in reducing PTSD symptoms as compared to a control group. The treatment was performed in two stages. The first aimed to explain the intervention, set up goals, and provide instructions for self-exposure. The second stage involved exposure in an earthquake simulator, explaining that this exposure was designed to increase the participant's sense of control. The simulator consisted of a small furnished house with a platform underneath that caused quakes of various intensities. Participants controlled the quaking (through a remote control) to initiate quakes and increase their intensity when they felt ready for that. If a participant's anxiety was more connected with the quakes, they were told to focus on the feeling, vision and sound of the movement. If anxiety was related to re-experiencing, they were encouraged to speak about the event as an imaginal exposure. The session with the simulator was finished when the participants felt in total control of their anxiety and fear. The mean duration of this second part of the intervention was 33 minutes. No sort of cognitive restructuring was used during the intervention. This treatment was found to be statistically significant and effective for the intervention group, despite the control group showing a 21% reduction in scores on the *Clinician-Administered PTSD Scale* (CAPS).

As compared to the previous study [17], the simulator exposure intervention with instructions for self-exposure was more effective in reducing PTSD symptoms than the intervention with instructions for self-exposure alone, with 79% and 59% of improvement, respectively, in treated groups. However, single-session interventions do not appear to be effective in cases of multiple co-morbidities [18].

Zang, Hunt and Cox [19] evaluated the efficacy of Narrative Exposure Therapy (NET) as a short-term treatment for PTSD in Chinese survivors from the Sichuan earthquake. Participants were stimulated to provide a chronological narrative of their lives focused on the trauma. This intervention is designed to help the cognitive process and habituation of emotional responses to the trauma. A comparison of pre-test, post-test and follow-up showed a statistically significant reduction of PTSD symptoms in the group receiving NET. Moreover, the treatment also offered benefits in co-morbidities, such as a reduction in depression and anxiety symptoms, gains which were maintained until two months after treatment. The authors concluded that NET was an effective treatment for earthquake survivors regarding reduction of PTSD symptoms and improvement of overall mental health.

### Non-randomized Controlled Studies

Goenjian, Karayan, Pynoos, Minassian, Najarian et al [15] evaluated the efficacy of a number of sessions of brief trauma-focused psychotherapy after the earthquake that hit Armenia in 1988. The intervention was initiated one and a half years after the earthquake. Treatment was based on five areas: reconstructing and reprocessing traumatic experiences and associated emotions; identifying and enhancing tolerance of reminders and links to traumatic experiences; enhancing acceptance, adapting to changes and losses related to the earthquake; mental reconstruction of deceased persons in the disaster; and promotion of normal development. The intervention group showed a statistically significant reduction of PTSD symptoms (intrusion, avoidance and autonomic hyperarousal) as compared to the control group. The trauma-focused psychotherapy showed good results in reduction of PTSD symptoms and prevention or worsening of depressive symptoms.

Goenjian, Walling, Steinberg, Karayan, Najarian et al [16] determined the effectiveness of trauma-focused therapy in improving PTSD and depressive symptoms, comparing treated and untreated adolescents. The intervention took place in a school setting and comprised four 90-minute group sessions and two 1-hour individual sessions. The most symptomatic students received up to 4 individual sessions and sessions with the family when indicated. The intervention used CBT techniques and was based on five areas mentioned above in the Goenjian, Karayan, Pynoos, Minassian, Najarian et al [15] study. Before treatment (15 years after the earthquake), the groups did not differ significantly in PTSD symptoms. However, 5 years after the earthquake, PTSD symptom scores (re-experiencing, avoidance and autonomic hyperarousal) were significantly lower in treated than in untreated students. Although the scores decreased in both groups, the reduction was three times greater in treated students. There was also a significant reduction in depressive symptoms in students who received treatment and an increase in those who did not.

Ferdos and Seyed-Hossein [24] assessed the effectiveness of a problem solving program in survivors of an earthquake in Iran in 2003. Training sessions of 2 hours each were held three times a week for one month and conducted by a therapist and a co-therapist. The first session was aimed at providing information about the intervention program and group rules. At the next three sessions the patients were taught coping strategies and trained in problem solving. At the fifth session training strategies were reviewed. At the sixth session control of intrusive thoughts was introduced. From the seventh to the twelfth sessions participants gave some feedback on the strategies chosen to cope with the problems, how effective they felt they were, and were encouraged to discuss the positive and negative aspects of the chosen strategies, preparing individuals to make their choices for problem solving by themselves. The experimental groups showed a significant increase in coping strategies skills focused on problem solving rather than on emotion, i.e. more effective coping strategies.

### Non Controlled Studies

Basoglu, Livanou, Salcioglu and Kalender [20] investigated with earthquake victims how brief a CBT intervention for PTSD could be, without undermining its effectiveness. The number of sessions for the intervention was not previously determined, the interval between sessions was about 16 days, and the treatment was finished when there was clinical improvement as observed by the therapist and patient. The treatment was conducted individually and in groups, and was divided in three steps. The first consisted in identifying the problems that emerged after the earthquake, such as avoidances, autonomic hyperarousal and re-experiencing symptoms. Pyschoeducation on the treatment came next. The third and last step of the treatment involved setting up goals and instructions for self-exposure to the avoidances raised in the first step. No cognitive restructuring was performed during treatment. The intervention was performed in a mean of 3–4 sessions. There was no difference between patients that were using or not using on psychiatric medication. A limitation of this study was the low number of participants in the follow-up evaluation, making it impossible to determine if the improvement in PTSD symptoms was maintained.

Giannopoulou, Dikaiakou and Yule [21] examined the effects of a cognitive-behavior intervention in a group of children with PTSD after the earthquake that hit Athens in 1999. Children that had another childhood-related comorbid psychopathology were not included in the study. The first session was a meeting with the parents to provide psychoeducation on the disorder and treatment. Children participated in six sessions, each including the session's agenda, a review of the previous week's and homework, psychoeducation and skill training, practice assisted by the therapist and setting homework for the next week. At the first session with the children (second session of the intervention), group rules were set up and PTSD symptoms were explained as a common reaction in those who undergo difficult or abnormal situations. At session two techniques for promoting a sense of control over trauma-related memories, nightmares and emotions were taught. The third session focused on symptoms of autonomic hyperarousal, and the children were taught how to identify reactions and to use progressive muscle relaxation and breathing techniques when they occur. At the fourth session avoidant behaviors were worked on by introducing the hierarchy of avoidances, and through the “fear thermometer” they could rank situations, places, people and memories they had been avoiding since the trauma. Sessions 5 and 6 were devoted to exposure, when the model of gradual exposure was explained and the participants were encouraged to perform imaginal exposure in the session. In vivo exposures were assigned as homework with the help of the parents. At the seventh session all that had been learned during the intervention as regards anxiety management and exposure was reinforced and summarized, with an emphasis on relapse prevention, as well as working on plans for the future. The intervention produced a statistically significant reduction in PTSD (intrusions, avoidances and autonomic hyperarousal) and depressive symptoms at the end of treatment and 18 months later. Of the 17 children who completed the treatment only 2 continued to meet the diagnosis of PTSD.

In the three studies described below we had to separate the intervention groups. The results of the studies were used separately, that is, each group was analysed as if it were an intervention group. We did not include them in the randomized or non-randomized categories because they did not have a group with no intervention that could be used as a control group. Even though the two studies were randomized studies, for the present review they were classified as open trials/non controlled studies, because the results of the intervention for each group were used separately.

Oflaz, Hatipoglu and Aydin [25] assessed the effectiveness of a psychoeducational intervention, including problem solving and coping strategies, as compared to medication and medication with psychoeducation group, in reducing PTSD symptoms in Turkish earthquake survivors. At the first session participants were interviewed about their feelings and beliefs about the traumatic experience. The second session and third session were dedicated to psychoeducation about the disorder, giving details on the nature of posttraumatic reactions. At the fourth session problem solving was introduced. At the fifth session the patients set up goals and were stimulated to put them in practice, in the interval between sessions. The sixth session was devoted to a review of what had been done and learned during the intervention. A statistically significant reduction in PTSD symptoms was observed in the medication with psychoeducation only group.

Jaycox, Cohen, Mannarino, Walker, Langley et al [22] identified students with PTSD symptoms from three schools following hurricane Katrina and offered them two interventions (Cognitive-Behavioral Intervention for Trauma in Schools – CBITS and Trauma-Focused Cognitive-Behavioral Therapy – TF-CBT). Both interventions used cognitive-behavioral strategies, such as psychoeducation, relaxation techniques, management of emotions, cognitive restructuring, narration of trauma and exposures. TF-CBT was conducted in 12 joint sessions, with child and parents, and was used only in a clinical context, while CBITS was delivered in groups in a school setting and only with the child, consisting of 10 group sessions and 1–3 individual sessions. Both groups had a statistically significant reduction in PTSD symptoms. Despite the reduction in PTSD symptoms, 37 children in the CBITS and one in the TF-CBT group still met the diagnostic criteria of PTSD at a 10-month follow-up. Family support was correlated with less impairment caused by the disorder.

Zhang, Feng, Xie, Xu and Chen [23] studied the curative effect of acupoint stimulation on the earthquake-caused PTSD in a group of patients. The subjects submitted to CBT were asked to describe the earthquake event (trauma) as they had experienced it, including the most horrifying scene, rescue of family members, pain sensation, and psychological and psychophysiological discomfort. As a result there were no dropouts, even though the patients suffered distress recalling the effects of the trauma. The authors conclude that CBT alone and CBT with acupoint stimulation are both effective in reducing PTSD symptoms, but suggest CBT in conjunction with acupoint stimulation be used in disaster-stricken areas, because it was found to be more effective than CBT alone.

## Discussion

### Main Findings

The efficacy of CBT for PTSD is well established as documented by systematic reviews [26], [27] and meta-analyses [28], [29]. The examination of the primary research on which reviews and meta-analyses are based, shows that samples from a diversity of traumas are used, particularly sex abuse, war and traffic accidents [30]–[33]. Few reviews attempt to focus on a specific trauma, such as Dossa and Hatem [29] on the use of CBT in adult women with PTSD who were victims of war violence, where 9 randomized studies were found. In the present review, in contrast, only 3 randomized studies were found. There are more randomized controlled trials.

As far as we know, this is the first systematic review focusing only on natural disasters. Of the 11 articles included here, all (3 randomized-control trials, 3 non-randomized controlled trials and 5 non controlled studies) reported a significant reduction of PTSD symptoms after treatment, suggesting efficacy of intervention. However, 10 of the 11 articles evaluated earthquake victims and only one evaluated victims of any other natural disaster, a hurricane, and no studies were found on landslides, forest fires, floods, volcanic eruptions, meteorite falls or tsunamis (for the latter, studies were found, but they did not meet the inclusion criteria of this review). Therefore, there is no evidence of the efficacy of CBT for victims of natural disasters other than earthquakes.

The restriction of samples to certain age groups precludes generalizing the claim of CBT efficacy in PTSD following natural disasters. Of the studies, 6 were with adults, 2 with adolescents and 2 with children, and one was with a mixed sample (ranging from 6 to 80 years). No study was found focusing solely on an elderly population, even though this is the group that is most vulnerable to post-disaster psychiatric morbidity [34]. Therefore, generalization of the efficacy outcome was partially precluded regarding age groups.

The randomized-controlled trials employed only the exposure technique as intervention and there was a significant reduction of PTSD symptoms in these studies. A number of independent groups of randomized-controlled trials around the world point to the efficacy of prolonged exposure therapy in the treatment of PTSD, with rapid change and maintenance of treatment gains over time [35]. The findings of the present review corroborate those of previous reviews [7], [26], [35], finding that exposure in its various forms (in vivo; quake platforms; narration of trauma) is effective in reducing PTSD symptoms.

### Methodological Issues

Considering that 225 million people are annually exposed to natural disasters [3], the existence of only 6 controlled studies evaluating the efficacy of CBT in patients with PTSD following this type of trauma shows a huge gap in the evidence-based literature. The existing studies involved only 742 people with PTSD, indicating the need for more research in the area, particularly with stronger methodologies, including more follow-ups (only 3 of 11 articles evaluated participants after one year of intervention) and randomized controlled trials so as to guarantee the efficacy of this therapy for PTSD related to natural disasters. Other authors have already warned about this paucity, taking into account the fact that natural disasters are one of the most studied events as a cause of PTSD, without there having been an equal focus on treatment.

### Limitations

One limitation of this review concerns the use of five electronic databases, even though they represent the main ones. No experts in the field, other than the authors of articles requested by e-mail, were consulted, leading to no information about non-published studies. Another limitation was the heterogeneity of studies, which made it impossible to perform a meta-analysis.

### Future Directions

We recommend the development of additional randomized controlled trials to validate the findings of this review, as already highlighted elsewhere [21], [27], as well as studies with more follow-ups, to investigate if the positive results reported for CBT use in reducing PTSD symptoms are maintained over time.

More studies with group interventions and using technological resources should be conducted, because natural disasters usually affect a large number of people. Of the studies covered in this review, four used group intervention [15], [16], [21], [22] and only one used technological resources [18]. A technological resource that could be used to reach more people, and even reach inaccessible places, is the Internet (through computers, cell phones and other portable devices), evaluating beforehand the viability of this according to the country stricken and resources available to the population. None of the articles reviewed here used such a resource, even though this type of intervention for PTSD is already possible [36], [37].

A large number of children develop PTSD after natural catastrophes [38]–[40]. It is crucial that these children be identified quickly and offered an effective treatment for the disorder [22]. However, there are few controlled studies about treatment for children and adolescents following disasters [15]. In this review we found only 2 non-randomized controlled studies and 3 open trials with children and adolescents in their samples.

Social and cultural adaptation of psychotherapy is particularly important as regards PTSD following natural disasters [27]. Most of the damage caused by natural disasters occurs in more populous regions, like Asia, with greater number of deaths and affected people [2]. The question of how to offer psychotherapy as developed in the West, with its proven efficacy, to a population with different perceptions of stress and strategies to deal with it, may be a challenging one [8], [34]? In order to adapt a typically Western treatment to an Eastern culture, we need to take into account the way they conceive mental health, and its services interventions should be adapted to local cultural and religious backgrounds. It is important that these populations do not view CBT as an imported approach, but as a proven technique that requires cultural adaptations for a better result [8]. Although this is already underway in recent studies [19], [23], [41], more studies should be conducted in order to get to know what is actually well accepted by the population (taking into account adherence and number of dropouts), as well as to evaluate if historical strategies of treatment of this population can also be beneficial in conjunction with CBT.

Another potential focus of studies in this area is the training of therapists to work in situations of natural disasters. Evidence-based treatments, including prolonged exposure therapy, are highly underutilized, resulting in unnecessary suffering, increased health costs, and work absenteeism [35]. Mclean and Foa [35] summarize the efforts, successes and challenges for disseminating prolonged exposure (PE) therapy. It was noted that permanent supervision of PE experts is a key element for successful treatment.

The results of this systematic review suggest the efficacy of CBT for treatment of PTSD following earthquakes, mainly with regard to the use of exposure techniques. However, in order for these results to be confirmed, we need further studies with more methodological rigor, and that include other types of natural disasters.

## Supporting Information

Checklist S1 **PRISMA Checklist used in this study.**
(DOC)
